# Differential Methylation in *APOE* (Chr19; Exon Four; from 44,909,188 to 44,909,373/hg38) and Increased Apolipoprotein E Plasma Levels in Subjects with Mild Cognitive Impairment

**DOI:** 10.3390/ijms20061394

**Published:** 2019-03-20

**Authors:** Oscar Mancera-Páez, Kelly Estrada-Orozco, María Fernanda Mahecha, Francy Cruz, Kely Bonilla-Vargas, Nicolás Sandoval, Esneyder Guerrero, David Salcedo-Tacuma, Jesús D. Melgarejo, Edwin Vega, Jenny Ortega-Rojas, Gustavo C. Román, Rodrigo Pardo-Turriago, Humberto Arboleda

**Affiliations:** 1 Department of Neurology, Faculty of Medicine, Universidad Nacional de Colombia, Bogotá ZC 57, Colombia; rpardot@unal.edu.co; 2 Neurosciences Research Group, Faculty of Medicine, Universidad Nacional de Colombia, Bogotá ZC 57, Colombia; kpestradao@unal.edu.co (K.E.-O.); Fjcruzs@unal.edu.co (F.C.); kjbonillav@unal.edu.co (K.B.-V.); edwin.alberto.vega@gmail.com (E.V.); Harboledag@unal.edu.co (H.A.); 3 Genetic Institute, Universidad Nacional de Colombia, Bogotá ZC 57, Colombia; mfmahechaa@unal.edu.co (M.F.M.); nicocardavid@hotmail.com (N.S.); emguerrerog@unal.edu.co (E.G.); drsalcedot@unal.edu.co (D.S.-T.); jesus.melgarejo1024@gmail.com (J.D.M.); jcortegar@unal.edu.co (J.O.-R.); 4 David Cabello International Alzheimer Disease Scholarship Fund, Houston Methodist Hospital, Houston, TX 77030, USA; 5 Center for Evidence to Implementation, Bogotá ZC 57, Colombia; 6 Health Technologies and Politics Assessment Group, Clinical Research Institute, Faculty of Medicine, Universidad Nacional de Colombia, Bogotá ZC 57, Colombia; 7 PhD Program in Clinical and Translational Science, Department of Translational Research and of New Surgical and Medical Technologies, University of Pisa, 56128 Pisa, Italy; 8 Laboratory of Neuroscience, University of Zulia, Maracaibo 4001, Venezuela; 9 Department of Neurology, Methodist Neurological Institute and the Institute for Academic Medicine Houston Methodist Research Institute, Houston Methodist Hospital, Houston, TX 77030, USA; Gcroman@houstonmethodist.org; 10 Weill Cornell Medical College, Department of Neurology, Cornell University, New York, NY 10065, USA; 11 Hospital Universitario Nacional de Colombia, Bogotá ZC 57, Colombia

**Keywords:** *APOE* gene, apolipoprotein E, DNA methylation, mild cognitive impairment, Hispanics

## Abstract

Background: Biomarkers are essential for identification of individuals at high risk of mild cognitive impairment (MCI) for potential prevention of dementia. We investigated DNA methylation in the *APOE* gene and apolipoprotein E (ApoE) plasma levels as MCI biomarkers in Colombian subjects with MCI and controls. Methods: In total, 100 participants were included (71% women; average age, 70 years; range, 43–91 years). MCI was diagnosed by neuropsychological testing, medical and social history, activities of daily living, cognitive symptoms and neuroimaging. Using multivariate logistic regression models adjusted by age and gender, we examined the risk association of MCI with plasma ApoE and *APOE* methylation. Results: MCI was diagnosed in 41 subjects (average age, 66.5 ± 9.6 years) and compared with 59 controls. Elevated plasma ApoE and *APOE* methylation of CpGs 165, 190, and 198 were risk factors for MCI (*p* < 0.05). Higher CpG-227 methylation correlated with lower risk for MCI (*p* = 0.002). Only CpG-227 was significantly correlated with plasma ApoE levels (correlation coefficient = −0.665; *p* = 0.008). Conclusion: Differential *APOE* methylation and increased plasma ApoE levels were correlated with MCI. These epigenetic patterns require confirmation in larger samples but could potentially be used as biomarkers to identify early stages of MCI.

## 1. Introduction

Mild Cognitive Impairment (MCI) affects 3–20% of individuals older than 65 years, with prevalence rates varying according to geographic regions [1,2,3,4]. Approximately 20% of elderly individuals with diagnosed MCI would develop dementia [5,6]. Interestingly, although Hispanics from Latin America (LA) have almost two-fold higher risk of developing Late-Onset Alzheimer’s disease (LOAD) than Caucasian North Americans [7,8], the rates of MCI reported in individuals from the United States (US) [9,10] are notably higher than among Hispanics in LA (20% vs. <10%) [11]. This could be attributed to underdiagnosis of MCI in many regions of LA and failure to identify individuals at high risk for dementia. Additionally, numerous vascular risk factors associated with both MCI and dementia occur in Hispanics at higher rates and often with insufficient treatment, including hypertension, diabetes mellitus, smoking, sedentary lifestyle, hyperhomocysteinemia, obesity and dyslipidemia [12,13,14,15,16]. Thus, from the public health perspective, it is critical to implement new strategies to identify subjects at high risk for MCI to prevent and/or delay the development of dementia in this highly susceptible population.

The study of genetic traits is important to investigate the early stages of complex diseases such as AD. In fact, the ApoE-ε4 variant has been demonstrated to be the major genetic risk factor for AD in the general population [17]. The apolipoprotein E (ApoE) has three isoforms, ApoE-ε2, ApoE-ε3, and ApoE-ε4, with direct genetic correspondence to the ε2, ε3, and ε4 alleles. Besides the allelic variant, increased plasmatic apolipoprotein E levels have been examined in relation to AD risk [18]. However, reduced plasma apolipoprotein E levels have been considered a marker of progression of cognitive impairment independently of the *APOE* genotype [19,20]. Moreover, subjects with different dementia types and with one or two copies of the ε4 allele of the *APOE* gene exhibit decreased expression levels of serum apolipoprotein E with regard to both earlier onset of symptoms and deposits of beta-amyloid plaques [21,22,23].

Epigenetic modifications such as DNA methylation at CpG sites within the genome influence protein expression levels [24]. Hypermethylated promoters are primarily associated with gene expression inhibition [25]; however, in some instances, hypermethylation has been associated with enhanced expression of some genes such as TREM2 in LOAD [26]. The *APOE* gene has a bimodal methylation structure, with a hypomethylated CpG promoter and with comparatively hypermethylated CpG sites located in the *APOE* exon 4 to 3′ UTR region. In AD brains, the *APOE* CpG sites are differentially methylated in both a tissue-specific and an *APOE* genotype-specific manner [27].

**Figure 1:**
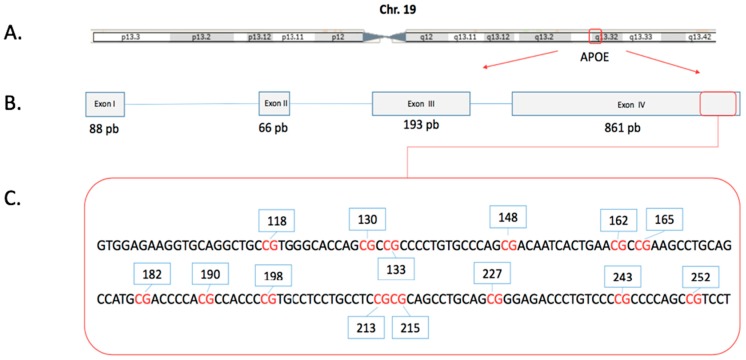
Schematic map of the human APOE gene. (**A**) The *APOE* gene contains four exons, is on the long arm of chromosome 19. (**B**) APOE gene structure (Red arrow) and the region evaluated in this study (Red box), (**C**). DNA sequence of the region evaluated by the BSP methodology, CpGs dinucleotides in red text and identification of each one in blue boxes. The genomic positions are described in Table 2.

Although the expression of *APOE* and its differential methylation levels in LOAD have been explored, there are no studies in subjects with MCI in LA describing the relationships between *APOE* methylation levels and apolipoprotein E differential expression. Therefore, we conducted this research study to estimate the DNA methylation levels for the *APOE* gene (Chr19; exon four; from 44,909,188 to 44,909,373) (Figure 1) and plasma levels of apolipoprotein E (ApoE) in a sample of LA subjects from Colombia with MCI; furthermore, we explored the relationships among *APOE* genotype, DNA methylation of the *APOE* gene and the risk of MCI.

## 2. Results

### 2.1. Baseline Characteristics

Of the total 100 participants evaluated, 41 had MCI and 59 were controls (Table 1). The mean age of the whole selected sample was 68.9 ± 9.5 years, and 71% (*n* = 71) were women with an age range of 43–91 years old. There was no statistically significant difference in the ApoE-ε4 distribution between MCI and controls.

### 2.2. Plasma ApoE Levels and APOE Methylation

Table 2 shows the genomic position of each CpG sites and compares the genotype traits between individuals with MCI and the normal control group. The plasma ApoE levels were higher among those with MCI (*p* < 0.001). *APOE* methylation of CpGs 118 (*p* = 0.009), 165 (*p* = 0.040), 190 (*p* = 0.045), 198 (*p* = 0.010) and 227 (*p* < 0.001) were lower in MCI participants (CpGs = 118, 165, 190, and 198) and only one was reversed (CpG-227). Comparisons between non-*APOE*-ε4 carriers and *APOE*-ε4 carriers (Table 3) showed that only CpG-148 was differently distributed (*p* = 0.003), being higher among *APOE*-ε4 carriers; the remaining CpG sites were similarly distributed (*p* > 0.05).

### 2.3. Plasma ApoE Levels and APOE Methylation Levels as Risk Factors for MCI

Logistic regression models adjusted by age and sex (Table 4) demonstrated that the increment on plasma ApoE levels (OR = 1.07; 95% CI = 1.02–1.13; *p* = 0.003), CpG-165 (OR = 1.20; 95% CI = 1.01–1.43; *p* = 0.045), and CpG-190 (OR = 1.52; 95% CI = 1.06–2.19; *p* = 0.042) can be considered risk factors for MCI. Higher CpG-227 methylation (OR = 0.49; 95% CI = 0.31–0.78; *p* = 0.002) correlated with lower risk for MCI. 

### 2.4. Correlation between Plasma ApoE Levels and APOE Methylation Levels

**Figure 2:**
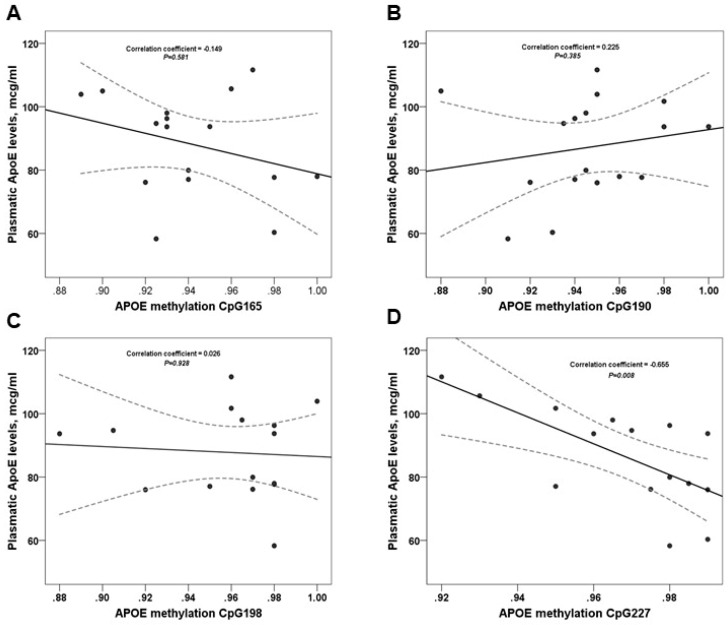
Correlation between Plasmatic ApoE levels and *APOE* Methylation CpGs. The correlation coefficient and *p* values for (**A**–**C**) were calculated using Pearson’s test; and for (**D**) we used the Spearman’s rank correlation.

The direct comparisons of plasma ApoE levels and *APOE* methylation are shown in Figure 2. We observed a trend for CpG-165 and CpG-19 but the association with plasma ApoE levels was not significant (*p* > 0.05). Moreover, we found a negative significant association between plasma ApoE levels and CpG-227 (*p* = 0.008).

## 3. Discussion

In the present study, we examined the association between plasma ApoE levels and *APOE* methylation in 14 CpGs in Chr19, exon IV; from 44,909,188 to 44,909,373 between participants with MCI and control subjects from Bogotá, Colombia, South America. Our key findings were: (i) individuals with MCI had increased plasma ApoE levels in contrast with healthy cognitive controls; (ii) rather than considering global methylation levels, we found that diverse *APOE* CpGs were differentially methylated when comparing participants with MCI and control subjects; (iii) after adjustment by age and sex, increments in ApoE plasma levels and CpG-165, CpG-190 and CpG-198 were found to be associated with increased risk of MCI, whereas lower CpG-227 methylation was related with lower risk; and (iv) only CpG-227 showed a significant correlation with plasma ApoE levels. Although confirmatory studies in larger samples are required, we suggest that assessment of MCI should include plasma ApoE levels and *APOE* methylation levels in order to identify individuals at high risk of developing dementia [28].

Previous studies have shown that decreased serum ApoE levels [28] and hypomethylation in the CpG-252 [26] (cg18799241) are risk factors for the development of dementia. We also found an inverse relationship in which higher plasma levels of ApoE were associated with MCI risk. Our findings might be due to differences in methylation patterns between cell types, with neurons holding higher global levels of DNA methylation [27] and with methylation variations in peripheral blood mononuclear cells related with shortening telomere length [29]. On the other hand, it should be noted that our study examined the possible pathophysiological process involved in a pre-dementia phase and thus our findings may suggest that high concentrations of ApoE would generate a more significant burden of amyloid beta deposition. This is supported by the fact that ApoE protein has a removal effect on amyloid beta [30]. However, this hypothesis cannot be verified with our current research model.

We report that serum ApoE and CpG regions were differentially methylated in MCI patients in contrast with the control group. We found both decreased and increased DNA methylation associated with MCI. Whether the increased DNA methylation of the APOE CpG-165, CpG-190 and CpG-198 is a cause or a consequence of cognitive decline remains to be studied. Foraker et al. [27] suggested an enhancer role of CGI that can be altered by DNA methylation and can modulate gene expression of both *APOE* and *TOMM40* with possible implications in ApoE expression and mitochondrial function. Additionally, these alterations in DNA methylation within genes that are essential for the mitochondrial function could contribute to structural changes in protein and mRNA instability [31]. Our findings support this view, as we found that CpG-227 was correlated with plasma ApoE levels. Despite no statistically significant association, CpG-165 and CpG-190 showed a tendency in relation to ApoE plasma levels.

Liu et al. [32] suggested that hypermethylation levels at multiple CpGs in the *APOE* genomic region are associated with delayed recall during cognitive aging. A previous study of our group [33] found an absence of differences in global *LINE-1* DNA methylation in LOAD subjects; however, this does not imply lack of alterations in DNA methylation for specific loci and their contribution to exonization events and lately in the epigenetic modifications of the landscape [31]. Consequently, global APOE DNA methylation can be useful to complement locus-specific subanalysis. In the same way, the ability to detect DNA methylation in patients with MCI could be enhanced by new approaches focused on specific cell-analysis, such as distinct cerebral cortex layers [27] and correlation with in vivo brain flow biomarkers [34,35,36,37,38]. 

Yu et al. [39] found that *APOE* CGI exhibits transcriptional enhancer or silencer activity, the mean percentage of methylation *APOE* CGI tends to be directly proportional with *APOE* expression, although it did not reach the cutoff value of statistical significance. 

To the best of our knowledge, we found no previous studies analyzing the methylation pattern in this specific locus in subjects with MCI and its correlation with *APOE* transcriptional activity; therefore, the underlying mechanisms of transcriptional regulation of *APOE* and correlation with CpG 227 will need to be studied in a larger sample of patients with higher statistical significance.

Developing countries do not usually have advanced diagnostic methods that can be implemented to identify patients at high risk for MCI. Thus, the study of peripheral blood DNA methylation promises to be a useful pre-clinical biomarker of MCI.

## 4. Materials and Methods

### 4.1. Study Design and Population Sample

Participants from a cohort of Colombian patients enrolled at the Memory Clinic of the National University of Colombia agreed to participate in this research. Inclusion criteria were: (i) individuals free of dementia at baseline assessment; and (ii) available data of plasma ApoE or APOE methylation levels. Exclusion criteria were: a history of schizophrenia, manic-depressive disorders, schizoaffective disorder, drug/dependence abuse, severe brain trauma or significant disability or unstable medical conditions (i.e., chronic renal failure, chronic hepatic disease, or severe pulmonary disease) and thyroid disease with no hormonal substitution. From a total of 100 participants, 41 had plasma ApoE levels, and 59 had APOE methylation data available (only 18 participants had both genetic phenotypes). Informed consent was obtained from both the participants and their closest relatives. This study was approved by the ethics committee of the National University of Colombia Act 011-107-15 (01/07/2015). All participants, or their closest relatives, gave written informed consent before participating in this study.

### 4.2. Medical Evaluation

A clinical neurological assessment was performed and all available data were registered, such as personal clinical history, mental and neurological examination, cognitive screening tests, neuropsychiatric inventory, functional scales and blood tests, e.g., lipid profile, glucose, thyroid function, vitamin B12 and folate levels, hepatic and renal function, serology VDRL, and complete metabolic panel. For those participants with abnormal cognitive tests, brain magnetic resonance image (MRI) was obtained and reviewed in consultation with our multidisciplinary team during follow-up.

### 4.3. Neuropsychological (NP) Evaluation

We used the Neuronorma Colombia (Neuronorm-Col) diagnostic NP battery for cognitive assessment [40,41,42,43,44,45,46,47,48]. Neuronorm-Col consists of language tests (Boston Naming Test and Token Test), visuoconstructive skills (Rey–Osterrieth Complex Figure), attention and executive functions (WAIS-III Digit Retention Tests, Corsi Cubes, Trail Making Tests A and B (TMT A and B), Digit–Symbol Test (SDMT), Stroop Color–Word Test, Tower of London test, Win-Dingo Card Sorting Test and Verbal Fluency), and memory (Free and Cued Selective Reminding Test) [40,41,42,43,44,45,46,47,48].

### 4.4. Diagnostic Classification of the Participants

#### 4.4.1. Mild Cognitive Impairment

MCI was diagnosed by consensus of a multidisciplinary group that included neurologists, neuropsychologists and neuroscientists, according to the criteria of Petersen et al. [4] modified from the Cognitive assessment test study described by Estrada-Orozco et al. [40]. Differential diagnosis of other related cognitive disorders was based on information from complete neuropsychological testing, medical and social history, activities of daily living, reported cognitive symptoms, and neuroimaging findings. Global cognitive functioning was assessed with the Neuronorm-Col diagnostic neuropsychological battery [40,41,42,43,44,45,46] and other functional scales [47,48]. NP criteria for MCI included scores 1.5–2.0 SD below education- and age-corrected values on at least two individual tests within a cognitive domain.

#### 4.4.2. Normal Performance in Healthy Subjects

Criteria for normal performance were: (1) no more than one test score lower than expected within a cognitive domain; and (2) no more than two scores lower than expected across domains, with the threshold corresponding to 1.0 standard deviation (SD) below age-adjusted control means. Moreover, medical and social history, activities of daily living, reported cognitive symptoms, and neuroimaging findings were reviewed to classify the subjects as healthy normal controls.

The control group was composed of cognitively healthy subjects who were selected based on the performances obtained in the screening scales and in the Neuronorma-Colombia battery, a neuropsychological battery normalized to our population in the context of the Spanish Multicenter Normative Studies (NEURONORMA project) [40,41,42,43,44,45,46] The cognitive domains evaluated in this battery were: attention (TMT A, TMT B, SDMT, Stroop Test, and Verbal and Direct Visual Span), memory (Grober & Buschke Test, Rey–Osterrieth Complex Figure Evocation), language (Boston Naming Test, Token Test, Verbal Fluency), visual-constructional skills (Rey–Osterrieth Complex Figure Copy), and executive functions (Verbal Phonological Fluency, Tower of London, Interference Stroop Test, and Wisconsin Card Sorting Test).

The following scores were considered normal for the screening tests: Montreal Cognitive Assessment (MOCA) [49], >24; INECO Frontal Screening (IFS) [50], >17.5; Yesavage scale [51], <5; Neuropsychiatric inventory (NPI) [52], <4; and Modified Lawton scale [47], 14–14. In the Neuropsychological tests [40,41,42,43,44,45,46,47,49,50,51,52,53,54], the cutoff point was one standard deviation (<1 SD) below the mean according to Petersen criteria [4]. Therefore, subjects with scores below the mean or <1 SD in two or more tests evaluating the same cognitive domain were discarded as controls [4].

### 4.5. DNA Extraction and Bisulfite Treatment

Genomic DNA was isolated from blood from patients and controls using the kit ReliaPrep Blood gDNA Miniprep System™ (A5082-PROMEGA, Fitchburg, WI, USA) following the protocol recommended by the company. DNA was Quantified in a spectrophotometer NanoDrop2000c ThermoScientific and then saved at 4 °C. Subsequently, the isolated DNA was bisulfite-converted using the EZ DN Methylation-Direct Kit (D5021–ZymmoResearch, Irvine, CA, USA). We then evaluated the methylation status of the APOE-CGI (APOE-ExonVI-CpG118 to CpG252) located in Chr19:44,909,188–44,909,373.

### 4.6. Bisulfite Sequencing PCR (BSP)

The APOE-CGI primers sequences from APOE-F-(5′-TGGAGAAGGTGTAGGTT-3′) and APOE-R-(5′-TTATTAAACTAAAATCCACCCC-3′) were designed following the parameters proposed by Clark et al. [55]. and modified from Tusnady et al. [56]. Each amplification reaction contained 200 ng of DNA, 20 pmol of each primer, 10% dimethyl sulfoxide, two mM dNTP, and 0.125 U of Taq DNA. Both primers were used in a final concentration of 200 nmol/L. Specificity of the assay for converted DNA was verified with the inclusion of unconverted genomic DNA as a control (non-converted DNA Human Methylated & Non-Methylated DNA set D5014 Zymo Research) [57]. Conditions for BSP assay were 95 °C for 5 min, 40 cycles of 95 °C for 30 s, 58.2 °C for 30 s, and 72 °C for 45 s and standardized in a thermocycler C1000 touch (Bio-Rad, Hercules, CA, USA) Then, the products were purified and sequencing in a ABI PRISM 3500 (Applied Biosystem, Foster City, CA, USA). Methylation percentage for each sample was calculated by analysis with ESME (Epigenetic Sequencing Methylation analysis Software, USA) [33,58].

### 4.7. Apolipoprotein Plasma Levels

Genotyping for APOE ε2, ε/3 and /ε4 allelic variants was determined as previously described, and ApoE plasma levels [26] were measured [28] using ELISA technique (Thermofisher-Invitrogen Human Apo E (AD2) ELISA Kit, CA, USA).

### 4.8. Statistical Analysis

The continuous variables are presented as mean and standard deviation (±) while categorical ones are summarized as frequencies and percentages (%). Global methylation level was calculated averaging each of the CpG sites. We compared the ApoE plasma and APOE methylation levels between participants with MCI and control group. Those APOE methylation CpGs following a non-parametric distribution were analyzed using the U-Mann–Whitney test for determining statistically significant differences; for the remaining traits, we used a *t*-student test. For categorical variables, we used Chi-square test. The plasma ApoE and APOE methylation levels were compared according to the APOE allelic variants. To determine the risk association of plasma ApoE levels and APOE methylation with MCI, we performed multivariate-adjusted models accounting for age and sex. Regression models were performed in those genetic phenotypes with significant average differences. Finally, CpG sites found as risk factors for MCI were correlated with plasma ApoE levels using Pearson’s correlation or Spearman’s rank tests when appropriated. Data management and statistical analysis were performed using SPSS version 23 (statistical package for social science). Statistical significance was accepted at *p* < 0.05 for two-tailed tests.

## 5. Limitations

The present study must be interpreted within the context of its potential limitations. First, the population sample presents a risk of selection bias because analyzed individuals attended a specialized care center for patients with memory complaints. However, selection bias was minimized by local radio and television announcements in an effort to recruit healthy subjects. Second, the small sample size limits the generalization of the findings. Third, although unlikely, it is possible that peripheral cellular populations with normal DNA methylation levels could mask the detection of more substantial methylation changes [59].

## 6. Conclusions

We found that, depending on the CpG region, decreased or increased DNA methylation levels, as well as increased plasma ApoE levels, are potential biomarkers for MCI. These findings might have implications for clinical practice given that these peripheral blood genetic phenotypes could be used for the early diagnosis of MCI. Moreover, if a high-risk profile for vascular cognitive impairment is identified [14], clinical intervention strategies to treat and control modifiable risk factors [16] associated with MCI progression can be intensively implemented to prevent or delay the development of dementia [14,16]. Further studies are needed to confirm these findings and to clarify the risk-association of DNA methylation from different tissues with MCI and neuropsychological profile, and to determine whether the clinical intervention of controlling modifiable risk factors found in dementia can modify the DNA methylation pattern and reduce the risk for MCI progression.

## Figures and Tables

**Table 1:** Baseline characteristics of the selected sample according to individuals with mild cognitive impairment and normal controls.

Baseline Characteristics	Whole Sample	MCI	Control	*p* Value *
	(*n* = 100)	(*n* = 41)	(*n* = 59)	
Demographic data				
Age, years				
Average	68.9 ± 9.5	66.5 ± 9.6	70.5 ± 9.1	0.029
Range	43–91	43–91	50–88	
Gender				0.008
Women, *n* (%)	71 (71.0)	35 (85.4)	36 (61.0)	
Men, *n* (%)	29 (29.0)	6 (14.6)	23 (39.0)	
Genetic traits				
*APOE*-ε4	25 (25.0)	10 (24.4)	15 (25.4)	0.999

MCI, mild cognitive impairment. * *p* value of comparison between controls and individuals with MCI. The Student *t*-test was used to calculate differences for average age and the Chi-square test for gender and *APOE*-ε4.

**Table 2:** Results of ApoE plasma levels and *APOE* methylation of CpGs sites and comparison between individuals with mild cognitive impairment and normal controls.

Variables	Genomic Position (hg38)	Whole Sample	MCI	Control	*p* Value *
		(*n* = 100)	(*n* = 41)	(*n* = 59)	
ApoE plasmatic Levels, mcg/mL	-	103.2 ± 26.5	113.8 ± 26.4	86.0 ± 15.7	<0.0001 ^†^
Global methylation	-	91.9 ± 3.0	92.8 ± 2.6	91.6 ± 3.1	0.154 ^†^
Methylation by CpG sites					
CpG118	chr19:44,909,208	85.6 ± 5.1	89.6 ± 4.1	84.7 ± 4.9	0.009 ^†^
CpG130	chr19:44,909,220	88.9 ± 7.1	89.0 ± 3.9	88.9 ± 7.6	0.484
CpG133	chr19:44,909,223	87.9 ± 10.1	85.7 ± 13.3	88.4 ± 9.3	0.620
CpG148	chr19:44,909,238	94.2 ± 4.2	94.9 ± 3.4	93.9 ± 4.4	0.255
CpG162	chr19:44,909,252	89.7 ± 6.1	90.9 ± 3.9	89.3 ± 6.7	0.361
CpG165	chr19:44,909,254	92 ± 5.2	94.5 ± 2.3	91.2 ± 5.6	0.040 ^†^
CpG182	chr19:44,909,272	95.2 ± 8.2	93.3 ± 11.7	95.9 ± 6.6	0.324
CpG190	chr19:44,909,280	93.8 ± 5.8	96.4 ± 2.3	93.0 ± 6.3	0.045 ^†^
CpG198	chr19:44,909,288	90.8 ± 7.9	96.2 ± 3.1	89.3 ± 8.2	0.01 ^†^
CpG213	chr19:44,909,303	95.1 ± 6.7	96.6 ± 6.8	94.7 ± 6.7	0.212
CpG215	chr19:44,909,305	90.7 ± 11.5	91.3 ± 12.9	90.5 ± 11.2	0.272
CpG227	chr19:44,909,317	97.7 ± 2.3	95.6 ± 2.5	98.4 ± 1.8	<0.0001
CpG243	chr19:44,909,333	91.9 ± 10.4	92.5 ± 11.9	91.6 ± 9.9	0.157
CpG252	chr19:44,909,342	90.4 ± 6.2	90.0 ± 6.1	90.6 ± 6.4	0.833

MCI, mild cognitive impairment. * *p* value of comparison between controls and MCI. ^†^ Student *t*-test was used to calculate differences; U-Mann–Whitney for variables with non-parametric distribution.

**Table 3:** Values of ApoE plasma levels and *APOE* methylation of CpGs sites according to *APOE* genotype.

Variables	Non-*APOE*-ε4 Carriers	*APOE*-ε4 Carriers	*p* Value *
	(*n* = 70)	(*n* = 25)	
ApoE plasma levels, mcg/mL	106.0 ± 31.3	103.0 ± 26.2	0.738 ^†^
Global methylation	92.2 ± 2.2	91.6 ± 3.3	0.557 ^†^
Methylation by CpG Sites			
CpG118	84.5 ± 6.0	85.8 ± 4.5	0.428 ^†^
CpG130	90.9 ± 6.5	88.0 ± 7.3	0.079
CpG133	84.8 ± 11.9	89.9 ± 7.0	0.087
CpG148	96.2 ± 2.9	93.3 ± 4.4	0.003
CpG162	90.8 ± 3.1	89.4 ± 7.0	0.447
CpG165	89.7 ± 4.3	92.6 ± 5.4	0.076 ^†^
CpG182	94.2 ± 10.1	95.4 ± 7.7	0.474
CpG190	95.0 ± 2.0	94.1 ± 3.1	0.316 ^†^
CpG198	91.1 ± 4.6	91.4 ± 5.6	0.860 ^†^
CpG213	95.6 ± 5.2	94.8 ± 7.4	0.872
CpG215	93.0 ± 8.1	89.6 ± 12.7	0.650
CpG227	97.5 ± 2.4	98.0 ± 2.1	0.482
CpG243	95.0 ± 3.6	90.0 ± 12.3	0.363
CpG252	89.9 ± 3.5	90.5 ± 7.3	0.200

* *p* Value of comparison between controls and individuals with MCI. ^†^ The Student *t*-test was implemented for calculating differences. The remaining quantitative variables were analyzed by using U-Mann–Whitney as they followed a non-parametric distribution.

**Table 4:** Adjusted logistic regression models to examine the association of plasma ApoE levels and *APOE* methylation with mild cognitive impairment.

Variables	Risk for Mild Cognitive Impairment		
	Odds Ratios	95% Confident Interval	*p* Value
Plasma ApoE levels	1.07	1.02–1.13	0.003
APOE methylation		-	
CpG 118	1.25	0.96–1.62	0.092
CpG 165	1.20	1.01–1.43	0.045
CpG 190	1.52	1.06–2.19	0.023
CpG 198	1.30	1.01–1.67	0.042
CpG 227 *	10.05	1.50–67.30	0.017

Models were adjusted by age and sex. * As CpG227 followed a non-parametric distribution, we divided it into four quartiles and <25th percentile was considered as the risk reference.

## References

[1] Gauthier S., Reisberg B., Zaudig M., Petersen R.C., Ritchie K., Broich K., Belleville S., Brodaty H., Bennett D., Chertkow H. (2006). Mild cognitive impairment. Lancet.

[2] Henao-Arboleda E., Aguirre-Acevedo D., Munoz C., Pineda D., Lopera F. (2008). Prevalence of mild cognitive impairment, amnestic-type, in a Colombian population. Rev. Neurol..

[3] Hänninen T., Hallikainen M., Tuomainen S., Vanhanen M., Soininen H. (2002). Prevalence of mild cognitive impairment: A population-based study in elderly subjects. Acta Neurol. Scand..

[4] Petersen R.C., Roberts R.O., Knopman D.S., Boeve B.F., Geda Y.E., Ivnik R.J., Smith G.E., Jack C.R. (2009). Mild cognitive impairment: Ten years later. Arch. Neurol..

[5] Manly J.J., Tang M.X., Schupf N., Stern Y., Vonsattel J.P.G., Mayeux R. (2008). Frequency and course of mild cognitive impairment in a multiethnic community. Ann. Neurol..

[6] Kaduszkiewicz H., Eisele M., Wiese B., Prokein J., Luppa M., Luck T., Jessen F., Bickel H., Mösch E., Pentzek M. (2014). Prognosis of mild cognitive impairment in general practice: Results of the German AgeCoDe study. Ann. Family Med..

[7] Farrer L.A., Cupples L.A., Haines J.L., Hyman B., Kukull W.A., Mayeux R., Myers R.H., Pericak-Vance M.A., Risch N., Van Duijn C.M. (1997). Effects of age, sex, and ethnicity on the association between apolipoprotein E genotype and Alzheimer disease: A meta-analysis. JAMA.

[8] Maestre G.E., Mena L.J., Melgarejo J.D., Aguirre-Acevedo D.C., Pino-Ramírez G., Urribarrí M., Chacon I.J., Chávez C.A., Falque-Madrid L., Gaona C.A. (2018). Incidence of dementia in elderly Latin Americans: Results of the Maracaibo Aging Study. Alzheimer’s Dement..

[9] Petersen R.C., Roberts R.O., Knopman D.S., Geda Y.E., Cha R.H., Pankratz V., Boeve B., Tangalos E., Ivnik R., Rocca W. (2010). Prevalence of mild cognitive impairment is higher in men The Mayo Clinic Study of Aging. Neurology.

[10] Plassman B.L., Langa K.M., Fisher G.G., Heeringa S.G., Weir D.R., Ofstedal M.B., Burke J.R., Hurd M.D., Potter G.G., Rodgers W.L. (2008). Prevalence of cognitive impairment without dementia in the United States. Ann. Intern. Med..

[11] Sosa A.L., Albanese E., Stephan B.C., Dewey M., Acosta D., Ferri C.P., Guerra M., Huang Y., Jacob K., Jimenez-Velazquez I.Z. (2012). Prevalence, distribution, and impact of mild cognitive impairment in Latin America, China, and India: A 10/66 population-based study. PLoS Med..

[12] Schargrodsky H., Hernández-Hernández R., Champagne B.M., Silva H., Vinueza R., Ayçaguer L.C.S., Touboul P.-J., Boissonnet C.P., Escobedo J., Pellegrini F. (2008). CARMELA: Assessment of cardiovascular risk in seven Latin American cities. Am. J. Med..

[13] Melgarejo J.D., Maestre G.E., Thijs L., Asayama K., Boggia J., Casiglia E., Hansen T.W., Imai Y., Jacobs L., Jeppesen J. (2017). Prevalence, treatment, and control rates of conventional and ambulatory hypertension across 10 populations in 3 continents. Hypertension.

[14] Knopman D., Boland L., Mosley T., Howard G., Liao D., Szklo M., McGovern P., Folsom A. (2001). Atherosclerosis Risk in Communities (ARIC) Study Investigators. Cardiovascular risk factors and cognitive decline in middle-aged adults. Neurology.

[15] Kivipelto M., Ngandu T., Fratiglioni L., Viitanen M., Kåreholt I., Winblad B., Helkala E.-L., Tuomilehto J., Soininen H., Nissinen A. (2005). Obesity and vascular risk factors at midlife and the risk of dementia and Alzheimer disease. Arch. Neurol..

[16] Román G.C., Mancera-Páez O., Bernal C. (2019). Epigenetic Factors in Late-Onset Alzheimer’s disease: *MTHFR* and *CTH* Gene Polymorphisms, Metabolic Trans-sulfuration and Methylation Pathways, and B Vitamins. Int. J. Mol. Sci..

[17] Tang M.-X., Stern Y., Marder K., Bell K., Gurland B., Lantigua R., Andrews H., Feng L., Tycko B., Mayeux R. (1998). The APOE-ε4 allele and the risk of Alzheimer disease among African Americans, whites, and Hispanics. JAMA.

[18] Taddei K., Clarnette R., Gandy S.E., Martins R.N. (1997). Increased plasma apolipoprotein E (apoE) levels in Alzheimer’s disease. Neurosci. Lett..

[19] Sullivan P., Han B., Liu F., Mace B., Ervin J., Wu S., Koger D., Paul S., Bales K. (2011). Reduced levels of human apoE4 protein in an animal model of cognitive impairment. Neurobiol. Aging.

[20] Song F., Poljak A., Crawford J., Kochan N.A., Wen W., Cameron B., Lux O., Brodaty H., Mather K., Smythe G.A. (2012). Plasma apolipoprotein levels are associated with cognitive status and decline in a community cohort of older individuals. PLoS ONE.

[21] Corder E.H., Saunders A.M., Strittmatter W.J., Schmechel D.E., Gaskell P.C., Small G., Roses A.D., Haines J., Pericak-Vance M.A. (1993). Gene dose of apolipoprotein E type 4 allele and the risk of Alzheimer’s disease in late onset families. Science.

[22] Liu N., Zhang K., Zhao H. (2008). Haplotype-association analysis. Adv. Genet..

[23] Chartier-Hariln M.-C., Parfitt M., Legrain S., Pérez-Tur J., Brousseau T., Evans A., Berr C., Vldal O., Roques P., Gourlet V. (1994). Apolipoprotein E, ε4 allele as a major risk factor for sporadic early and late-onset forms of Alzheimer’s disease: Analysis of the 19q13. 2 chromosomal region. Hum. Mol. Genet..

[24] Ciceri F., Rotllant D., Maes T. (2017). Understanding epigenetic alterations in Alzheimer’s and Parkinson’s disease: Towards targeted biomarkers and therapies. Curr. Pharm. Des..

[25] Bae M.G., Kim J.Y., Choi J.K. (2016). Frequent hypermethylation of orphan CpG islands with enhancer activity in cancer. BMC Med. Genomics.

[26] Shao Y., Shaw M., Todd K., Khrestian M., D’Aleo G., Barnard P.J., Zahratka J., Pillai J., Yu C.-E., Keene C.D. (2018). DNA methylation of TOMM40-APOE-APOC2 in Alzheimer’s disease. J. Hum. Genet..

[27] Foraker J., Millard S.P., Leong L., Thomson Z., Chen S., Keene C.D., Bekris L.M., Yu C.-E. (2015). The APOE gene is differentially methylated in Alzheimer’s disease. J. Alzheimer’s Dis..

[28] Rasmussen K.L., Tybjærg-Hansen A., Nordestgaard B.G., Frikke-Schmidt R. (2015). Plasma levels of apolipoprotein E and risk of dementia in the general population. Ann. Neurol..

[29] Paul L., Cattaneo M., D’Angelo A., Sampietro F., Fermo I., Razzari C., Fontana G. (2009). Telomere length in peripheral blood mononuclear cells is associated with folate status in men in me. J. Nutr..

[30] Cramer P.E., Cirrito J.R., Wesson D.W., Lee C.D., Karlo J.C., Zinn A.E., Casali B.T., Restivo J.L., Goebel W.D., James M.J. (2012). ApoE-directed therapeutics rapidly clear β-amyloid and reverse deficits in AD mouse models. Science.

[31] Larsen P.A., Lutz M.W., Hunnicutt K.E., Mihovilovic M., Saunders A.M., Yoder A.D., Roses A.D. (2017). The Alu neurodegeneration hypothesis: A primate-specific mechanism for neuronal transcription noise, mitochondrial dysfunction, and manifestation of neurodegenerative disease. Alzheimer’s Dement..

[32] Liu J., Zhao W., Ware E.B., Turner S.T., Mosley T.H., Smith J.A. (2018). DNA methylation in the APOE genomic region is associated with cognitive function in African Americans. BMC Med. Genomics.

[33] Hernández H.G., Mahecha M.F., Mejía A., Arboleda H., Forero D.A. (2014). Global long interspersed nuclear element 1 DNA methylation in a Colombian sample of patients with late-onset Alzheimer’s disease. Am. J. Alzheimer’s Dis. Other Dement..

[34] Du A., Jahng G., Hayasaka S., Kramer J., Rosen H., Gorno-Tempini M., Rankin K., Miller B., Weiner M., Schuff N. (2006). Hypoperfusion in frontotemporal dementia and Alzheimer disease by arterial spin labeling MRI. Neurology.

[35] Tosun D., Schuff N., Jagust W., Weiner M.W., Initiative A.s.D.N. (2016). Discriminative power of arterial spin labeling magnetic resonance imaging and 18F-fluorodeoxyglucose positron emission tomography changes for amyloid-β-positive subjects in the Alzheimer’s disease continuum. Neurodegener. Dis..

[36] Fällmar D., Haller S., Lilja J., Danfors T., Kilander L., Tolboom N., Egger K., Kellner E., Croon P.M., Verfaillie S.C. (2017). Arterial spin labeling-based Z-maps have high specificity and positive predictive value for neurodegenerative dementia compared to FDG-PET. Eur. Radiol..

[37] Musiek E.S., Chen Y., Korczykowski M., Saboury B., Martinez P.M., Reddin J.S., Alavi A., Kimberg D.Y., Wolk D.A., Julin P. (2012). Direct comparison of fluorodeoxyglucose positron emission tomography and arterial spin labeling magnetic resonance imaging in Alzheimer’s disease. Alzheimer’s Dement..

[38] Wolk D.A., Detre J.A. (2012). Arterial spin labeling MRI: An emerging biomarker for Alzheimer’s disease and other neurodegenerative conditions. Curr. Opin. Neurol..

[39] Yu C.-E., Cudaback E., Foraker J., Thomson Z., Leong L., Lutz F., Gill J.A., Saxton A., Kraemer B., Navas P. (2013). Epigenetic signature and enhancer activity of the human APOE gene. Hum. Mol. Genet..

[40] Estrada-Orozco K., Bonilla-Vargas K., Cruz F., Mancera O., Ruiz M., Alvarez L., Pardo R., Arboleda H. (2018). Cognitive Assessment Test: Validation of a Short Cognitive Test for the Detection of Mild Cognitive Disorder. Int. J. Alzheimer’s Dis..

[41] Espitia Mendieta D. (2017). Funciones Ejecutivas en el Envejecimiento Normal: Datos Normativos Con La Batería Neuronorma. Master’s Thesis.

[42] Peña-Casanova J., Quiñones-Ubeda S., Gramunt-Fombuena N., Quintana-Aparicio M., Aguilar M., Badenes D., Cerulla N., Molinuevo J.L., Ruiz E., Robles A. (2009). Spanish Multicenter Normative Studies (NEURONORMA Project): Norms for verbal fluency tests. Arch. Clin. Neuropsychol..

[43] Sánchez-Benavides G., Peña-Casanova J., Casals-Coll M., Gramunt N., Manero R.M., Puig-Pijoan A., Aguilar M., Robles A., Antúnez C., Frank-García A. (2016). One-Year Reference Norms of Cognitive Change in Spanish Old Adults: Data from the NEURONORMA Sample. Arch. Clin. Neuropsychol..

[44] Aranciva F., Casals-Coll M., Sánchez-Benavides G., Quintana M., Manero R.M., Rognoni T., Calvo L., Palomo R., Tamayo F., Peña-Casanova J. (2012). Spanish normative studies in a young adult population (NEURONORMA young adults Project): Norms for the Boston Naming Test and the Token Test. Neurología.

[45] Peña-Casanova J., Quiñones-Ubeda S., Gramunt-Fombuena N., Quintana M., Aguilar M., Molinuevo J.L., Serradell M., Robles A., Barquero M.S., Payno M. (2009). Spanish Multicenter Normative Studies (NEURONORMA Project): Norms for the Stroop color-word interference test and the Tower of London-Drexel. Arch. Clin. Neuropsychol..

[46] Cruz-Sanabria F., Bonilla-Vargas K., Estrada K., Mancera O., Vega E., Guerrero E., Ortega-Rojas J., Mahecha María F., Romero A., Montañés P. (2018). Análisis de desempeños cognitivos y polimorfismos en SORL, PVRL2, CR1, TOMM40, APOE, PICALM, GWAS_14q, CLU y BIN1 en pacientes con trastorno neurocognitivo leve y en sujetos cognitivamente sanos. Neurología.

[47] Gutiérrez C.A.C., Eslava D.L.M., Gavilán P.R., Ríos P.M. (2010). Cambios en las actividades instrumentales de la vida diaria en la Enfermedad de Alzheimer. Acta Neurol. Colomb..

[48] Jefferson A.L., Byerly L.K., Vanderhill S., Lambe S., Wong S., Ozonoff A., Karlawish J.H. (2008). Characterization of activities of daily living in individuals with mild cognitive impairment. Am. J. Geriatr. Psychiatry.

[49] Gil L., Ruiz de Sánchez C., Gil F., Romero S.J., Pretelt Burgos F. (2015). Validation of the Montreal Cognitive Assessment (MoCA) in Spanish as a screening tool for mild cognitive impairment and mild dementia in patients over 65 years old in Bogotá, Colombia. Int. J. Geriatr. Psychiatry.

[50] Romero-Vanegas S., Romero-Vanegas S., Vargas-Gonzalez J.C., Arboleda H., Lopera F., Pardo R. (2014). Validation of the ineco frontal screening in a colombian population. Alzheimer’s Dement..

[51] Angulo C.B.G., Arias A.C. (2011). Escala de Yesavage para Depresión Geriátrica (GDS-15 y GDS-5) estudio de la consistencia interna y estructura factorial. Univ. Psychol..

[52] Cummings J.L., Mega M., Gray K., Rosenberg-Thompson S., Carusi D.A., Gornbein J. (1994). The Neuropsychiatric Inventory: Comprehensive assessment of psychopathology in dementia. Neurology.

[53] Peña-Casanova J., Casals-Coll M., Quintana M., Sánchez-Benavides G., Rognoni T., Calvo L., Palomo R., Aranciva F., Tamayo F., Manero R.M. (2012). Estudios normativos españoles en población adulta joven (Proyecto NEURONORMA jóvenes): Métodos y características de la muestra. Neurología.

[54] Schinka J.A., Loewenstein D.A., Raj A., Schoenberg M.R., Banko J.L., Potter H., Duara R. (2010). Defining Mild Cognitive Impairment: Impact of Varying Decision Criteria on Neuropsychological Diagnostic Frequencies and Correlates. Am. J. Geriatr. Psychiatry.

[55] Clark S.J., Statham A., Stirzaker C., Molloy P.L., Frommer M. (2006). DNA methylation: Bisulphite modification and analysis. Nat. Protoc..

[56] Tusnady G.E., Simon I., Varadi A., Aranyi T. (2005). BiSearch: Primer-design and search tool for PCR on bisulfite-treated genomes. Nucleic Acids Res..

[57] Hernández H.G., Tse M.Y., Pang S.C., Arboleda H., Forero D.A. (2013). Optimizing methodologies for PCR-based DNA methylation analysis. Biotechniques.

[58] Lewin J., Schmitt A.O., Adorján P., Hildmann T., Piepenbrock C. (2004). Quantitative DNA methylation analysis based on four-dye trace data from direct sequencing of PCR amplificates. Bioinformatics.

[59] Mastroeni D., Grover A., Delvaux E., Whiteside C., Coleman P.D., Rogers J. (2010). Epigenetic changes in Alzheimer’s disease: Decrements in DNA methylation. Neurobiol. Aging.

